# Self-Compassion, Pain Centrality, and Trust in Physicians Among Chinese Patients with Chronic Pain: A Cross-Sectional Study

**DOI:** 10.3390/healthcare14203383

**Published:** 2026-10-10

**Authors:** Chen Gong, Yijiang Song, Haiping Hu, Baoyu Bai

**Affiliations:** 1 Department of Psychology, Academy of Advanced Interdisciplinary Studies, Wuhan University, Wuhan 430072, China; 2 Department of Acupuncture and Tuina, Ningxia Hospital of Integrated Traditional Chinese and Western Medicine, 499 Huaiyuan East Road, Xixia District, Yinchuan 750021, China; 3 School of Nursing, Wuhan University, 115 Donghu Road, Wuchang District, Wuhan 430071, China

**Keywords:** chronic pain, self-compassion, pain centrality, trust, physician–patient relations

## Abstract

**Background/Objectives**: Patients’ trust in physicians is important for treatment adherence and health outcomes in chronic pain, yet patients’ psychological traits remain underexamined. Self-compassion involves responding to personal suffering with self-kindness, a sense of common humanity, and mindful awareness. This study examined the association between self-compassion and trust in physicians among Chinese patients with chronic pain and explored the indirect association through pain centrality. **Methods**: A cross-sectional survey of 376 eligible hospitalized patients with chronic pain was conducted at a tertiary hospital in Yinchuan, Ningxia Hui Autonomous Region, China. The primary analyses included 367 participants with complete data on the study variables. Participants completed measures of self-compassion, pain centrality, and trust in physicians. Pearson correlations were calculated, and a bootstrap analysis of the indirect association was conducted while controlling for age, sex, 7-day average pain intensity, and log-transformed pain duration. **Results**: Self-compassion was positively associated with trust in physicians and negatively associated with pain centrality. Pain centrality was negatively associated with trust in physicians, and an indirect association through pain centrality was observed (estimate = 0.054, bootstrap standard error = 0.027, 95% confidence interval [0.0004, 0.1093]), while the direct association remained statistically significant. **Conclusions**: Self-compassion may represent a positive psychological resource relevant to trust in physicians, and pain centrality may be statistically relevant to this association. These findings support further investigation of both constructs in person-centered chronic pain assessment and care.

## 1. Introduction

Trust in physicians refers to patients’ willingness, in medical contexts characterized by uncertainty and dependence, to believe that physicians will act in their best interests. It is an important component of the patient–physician relationship and reflects patients’ cognitive and affective judgments regarding physicians’ competence, benevolence, and reliability [1]. In China, physician–patient conflicts have become an increasing concern, and a national survey reported that 66% of participating physicians had experienced medical disputes of varying degrees [2]. Within this context, patient–physician relationships and medical decision-making in China may also be shaped by cultural expectations concerning physician authority, relational hierarchy, and family-oriented decision-making [3,4]. These expectations may be relevant to how patients evaluate physicians’ expertise and benevolence, although cultural influences on trust were not directly examined in the present study. Low trust in physicians may reduce patients’ treatment adherence [5], weaken their confidence in the healthcare system [6], and increase the risk of conflict. Accordingly, understanding trust in physicians requires attention not only to characteristics of physicians and healthcare institutions but also to patient characteristics that may shape how illness and clinical interactions are experienced and interpreted.

Previous research has identified both physician-related and patient-related correlates of trust in physicians. Physician-related factors include professional reputation [7], communication skills, empathy, and hospital environment [8]. At the patient level, substantial attention has been given to sociodemographic and structural correlates, including educational attainment [9], medical insurance coverage [10], and socioeconomic status [11]. By comparison, patients’ positive psychological resources and pain-related self-representations have received less attention.

These gaps may be especially relevant in chronic pain. Chronic pain often involves prolonged treatment, repeated clinical encounters, and uncertainty regarding symptom course and treatment response [5]. Because pain is subjective and may not be fully reflected by objective indicators, patients’ psychological resources and pain-related self-representations may be particularly relevant to how healthcare relationships are perceived. This may be especially salient in inpatient settings, where active treatment and repeated interactions with physicians may make trust in physicians particularly relevant. Much of the existing literature on these processes, however, has been conducted in Western populations. Cultural norms may shape how pain is interpreted, expressed, and communicated during medical encounters [12], potentially influencing patients’ expectations of physicians and evaluations of care. These issues remain insufficiently examined among Chinese hospitalized patients with chronic pain.

Taken together, these considerations provide a basis for examining whether self-compassion, an intrapersonal orientation toward personal suffering, may also be relevant to interpersonal judgments in healthcare relationships characterized by uncertainty and dependence.

Self-compassion refers to responding to personal suffering and perceived inadequacy with kindness, balanced awareness, and recognition that suffering is part of the common human experience. It consists of three core components: self-kindness, common humanity, and mindfulness [13]. As a positive psychological resource, self-compassion may reduce self-criticism and psychological distress while promoting adaptive emotion regulation and coping [13,14]. In clinical settings, these capacities may help patients manage illness-related distress and approach difficult treatment experiences with less self-criticism and emotional overinvolvement. Such a more balanced orientation may also be relevant to how patients interpret uncertainty and interpersonal experiences in clinical relationships. Although direct evidence remains limited, self-compassion has been positively associated with interpersonal trust in both adolescent and adult samples [15,16]. Even though interpersonal trust is conceptually distinct from trust in physicians, these findings provide indirect support for examining whether self-compassion is also associated with trust in healthcare relationships characterized by vulnerability and dependence. Based on this theoretical and empirical framework, we hypothesize:

**H1.**  *Self-compassion will be positively associated with trust in physicians among Chinese patients with chronic pain.*

The proposed indirect association involves two component associations. First, we consider the association between self-compassion and pain centrality. Pain centrality refers to the extent to which chronic pain becomes central to an individual’s daily life, self-understanding, and identity [17,18]. We focused on pain centrality because, unlike pain intensity, pain interference, pain catastrophizing or pain acceptance, which primarily reflect symptom severity, functional impact, or psychological responses to pain, it captures the extent to which pain becomes incorporated into patients’ broader sense of self and identity. This makes it conceptually relevant to self-compassion, which concerns how individuals relate to their own suffering. Greater pain centrality has been associated with poorer pain acceptance and psychological adjustment [19]. Of particular relevance, the mindfulness component of self-compassion involves balanced awareness rather than overidentification with painful thoughts and emotions [13,20]. In chronic pain, this orientation may help patients acknowledge pain without allowing it to define the self completely or displace other valued roles and activities. Although the direct association between self-compassion and pain centrality has received little empirical attention, related evidence supports this possibility. Previous studies have found that greater self-compassion is associated with higher pain acceptance, greater engagement in valued activities, and more flexible coping [21,22]. Related intervention research has also reported improvements in pain acceptance and pain interference following mindful self-compassion training [23]. These findings provide indirect support for examining whether greater self-compassion is associated with lower pain centrality.

Second, we consider the association between pain centrality and trust in physicians. Physicians’ expressions of care, respect, empathy, and patient-centered communication during clinical interactions provide important bases for patients’ judgments of physicians’ benevolence, competence, and reliability [24]. Patients with chronic pain often rely on their own accounts to communicate their pain experiences to healthcare professionals. Whether their pain is believed, understood, and taken seriously by physicians therefore constitutes an important relational experience in clinical encounters [25,26]. However, people with chronic pain frequently report stigmatizing or invalidating responses, including having their pain disbelieved, discounted, or insufficiently understood by healthcare professionals [27,28]. When pain occupies a more central position in patients’ lives and identities, physicians’ responses to pain may therefore carry greater weight in evaluations of the clinical relationship. Although direct evidence linking pain centrality to trust in physicians is lacking, related findings provide indirect support. Previous studies have shown that feeling carefully listened to by physicians and perceiving greater physician empathy are both associated with higher trust in physicians [29,30]. These findings provide adjacent evidence that relational cues surrounding patients’ pain experiences may be relevant to trust in physicians, particularly when pain is highly central to the self. We therefore propose that pain centrality is negatively associated with trust in physicians.

Taken together, these two component associations provide the basis for examining an indirect statistical association through pain centrality, without implying that pain centrality is the sole factor relevant to trust in physicians. Accordingly, we hypothesize:

**H2.**  *Self-compassion will be indirectly associated with greater trust in physicians through lower pain centrality, while retaining a direct association with trust in physicians.*

The present cross-sectional study examined these hypothesized associations among hospitalized Chinese patients with chronic pain.

## 2. Materials and Methods

### 2.1. Design

This cross-sectional observational study was reported in accordance with the Strengthening the Reporting of Observational Studies in Epidemiology (STROBE) statement.

### 2.2. Sample Size

An a priori power analysis was conducted using G*Power version 3.1.9.7 for a fixed-model linear multiple regression framework. Assuming a medium effect size (*f^2^* = 0.15), an alpha level of 0.05, a statistical power of 0.90, and six predictors, the estimated minimum sample size was 123. This calculation was used for general sample-size planning within the regression framework; no prospective power analysis specifically targeting the indirect association was conducted.

### 2.3. Procedures and Participants

The study was conducted from February to May 2025 in the Department of Pain Medicine at Ningxia Hospital of Integrated Traditional Chinese and Western Medicine, a tertiary hospital in Yinchuan, Ningxia Hui Autonomous Region, China. Hospitalized patients with chronic pain were recruited using convenience sampling. For study eligibility, chronic pain was operationalized as a clinically diagnosed pain condition lasting for at least 3 months; no separate screening questionnaire was used to establish chronic pain status. Additional inclusion criteria were as follows: (1) sufficient cognitive and literacy abilities to understand and complete the questionnaire; (2) no severe language or hearing impairment that would interfere with questionnaire completion; (3) no documented diagnosis of a psychiatric disorder in the medical record; and (4) willingness to provide informed consent. Medical comorbidity, disability, and terminal illness were not exclusion criteria. In the final sample, some participants had medical comorbidities, whereas no participants were recorded as having a disability or terminal illness. Psychiatric exclusion was determined based on diagnoses documented in the medical record, and patients whose current clinical condition did not permit independent questionnaire completion were not invited to participate. Because the study relied on psychological self-report questionnaires, the psychiatric exclusion criterion was intended to reduce potential measurement heterogeneity associated with psychiatric comorbidity.

After providing written informed consent, participants received standardized instructions, and ward nurses assisted with questionnaire distribution. Participants were informed that there were no right or wrong answers and were encouraged to respond honestly according to their actual experiences. Questionnaire responses were kept confidential, and participants completed the questionnaires independently. The study measures were administered in a fixed order for all participants, and completed questionnaires were collected on site.

The numbers of patients initially approached and those who declined participation were not systematically recorded; therefore, a response rate could not be calculated. A total of 408 questionnaires were returned. Twenty-six participants were excluded because their reported pain duration was less than 3 months, and six were excluded for incorrectly answering both embedded attention-check items, leaving 376 participants who met the eligibility and data-quality criteria. Of these, 367 had complete data on all variables included in the primary analyses and comprised the final analytic sample. Specific reasons for hospitalization were not systematically recorded; therefore, we could not determine whether admission was primarily for pain management or for other medical conditions.

### 2.4. Measures

***Demographic and clinical information questionnaire.*** Participants reported their age, sex, marital status, place of residence, living arrangement, educational attainment, employment status, and monthly personal income. Clinical information collected for sample characterization included medical comorbidity, pain pattern, and pain location.

***Self-compassion.*** The Self-Compassion Scale was originally developed by Neff [14] and adapted into Chinese by Gong et al. [31]. The scale consists of 12 items across three dimensions: self-kindness (3 items; e.g., “I’m tolerant of my own flaws and inadequacies”), common humanity (4 items; e.g., “I try to see my failings as part of the human condition”), and mindfulness (5 items; e.g., “When something upsets me, I try to keep my emotions in balance”). Although the Chinese 12-item adaptation was initially developed in adolescents, subsequent studies have applied the same three-component version in Chinese adult samples and have analyzed self-compassion as an overall construct [32,33]. Items were rated on a 5-point Likert scale ranging from 1 *(never)* to 5 *(always)*. An overall mean score across the 12 items was calculated to represent global self-compassion, with higher scores indicating greater self-compassion. Cronbach’s α was 0.690 for the global self-compassion score in the present study.

***Pain centrality.*** The Centrality of Pain Scale [17], validated in Chinese by Wang et al. [34], is a 10-item unidimensional measure (e.g., “Pain controls my life,” “My pain defines who I am”). Items were rated on a 5-point Likert scale ranging from 1 *(strongly disagree)* to 5 *(strongly agree)*. A mean score was calculated, with higher scores indicating greater pain centrality. Cronbach’s α was 0.835 in the present study.

***Trust in physicians.*** The Chinese version of the Wake Forest Physician Trust Scale (WFPTS) was developed by Dong and Bao [35]. The scale consists of 10 items across two dimensions: benevolence (5 items; e.g., “My doctor is thorough and considerate”) and competence (5 items; e.g., “I feel the treatment plan chosen by my doctor is the most appropriate for me”). The questionnaire retained the wording of the validated Chinese version, and no additional instruction was provided to designate a specific physician as the referent. Items were rated on a 5-point Likert scale ranging from 1 *(strongly disagree)* to 5 *(strongly agree)*. An overall mean score across the 10 items was calculated to represent global trust in physicians, consistent with previous studies using the Chinese 10-item WFPTS as an overall trust measure [35,36,37]. Higher scores indicated greater trust in physicians. Cronbach’s α was 0.807 in the present study.

***Pain intensity and pain duration.*** Pain intensity was assessed using a single-item 11-point numerical rating scale (NRS), an established measure of pain intensity in chronic pain research [38,39,40]. Participants rated their average pain intensity during the previous 7 days from 0 (*no pain*) to 10 (*the worst pain imaginable*). For the primary analyses, this 7-day average pain rating was used as the pain-intensity indicator, with higher scores indicating greater pain intensity. Current pain intensity, average pain intensity during the previous six months, and worst pain intensity during the previous six months were also collected and were examined as alternative pain-intensity indicators in the sensitivity analyses. Pain duration was assessed using participants’ self-reported duration of pain in months.

### 2.5. Data Analysis

Descriptive statistics and Pearson correlations were computed using IBM SPSS Statistics version 26. Potential common method variance was explored using Harman’s single-factor test, in which all items from the three primary measures were entered into an unrotated exploratory factor analysis and the number of factors with eigenvalues greater than 1, the variance explained by the first factor, and their cumulative explained variance were inspected.

Before the primary analysis, diagnostic analyses were conducted for both regression equations. Linearity, homoscedasticity, residual distribution, multicollinearity, and potentially influential observations were examined using residual plots, variance inflation factors, studentized deleted residuals, leverage values, and Cook’s distances. Potential nonlinear relationships among the core variables were additionally examined using exploratory quadratic terms for self-compassion and pain centrality. Observations with an absolute studentized deleted residual greater than 3, leverage exceeding 2*p*/*n* (where *p* denotes the number of estimated regression parameters including the intercept), or a Cook’s distance greater than 1 were flagged for further inspection rather than automatically excluded.

The hypothesized indirect association was tested using Model 4 of the PROCESS macro version 4.1 for SPSS [41], with self-compassion specified as X, pain centrality as M, and trust in physicians as Y. Age, sex, average pain intensity during the previous 7 days, and log-transformed pain duration were included as covariates based on the prior literature and their theoretical and clinical relevance to the focal associations [42,43]. Because raw pain duration was substantially positively skewed, it was transformed as ln(pain duration + 1) before inclusion in the primary model. The indirect association was evaluated using 5000 bootstrap samples and a 95% bootstrap confidence interval. Because the data were cross-sectional, the PROCESS model was interpreted as a test of indirect statistical association rather than as evidence of a causal mechanism. Unstandardized regression coefficients, standard errors, and 95% confidence intervals are reported in the regression table.

Sensitivity analyses examined the extent to which the indirect association depended on covariate specification, the treatment of pain duration, and the operationalization of pain intensity. First, the sensitivity of the indirect association to covariate selection was examined using models with (a) no covariates, (b) age and sex as demographic covariates, and (c) age, sex, average pain intensity during the previous 7 days, and log-transformed pain duration as the primary covariate set. Second, the fully adjusted model was repeated using raw pain duration in place of log-transformed pain duration. Third, the primary model was repeated after replacing average pain intensity during the previous 7 days with each of three alternative pain-intensity indicators: current pain intensity, average pain intensity during the previous six months, and worst pain intensity during the previous six months. All statistical tests were two-tailed, with α set at 0.05.

## 3. Results

### 3.1. Participant Characteristics

A total of 376 participants met the eligibility and data-quality criteria. Their demographic and clinical characteristics are summarized in Table 1. The mean age was 44.00 years (SD = 14.12), and 46.0% of participants with available sex data were male. The median pain duration was 27 months (IQR = 13–60; range = 3–600 months), and the mean 7-day average pain intensity was 4.14 (SD = 2.06). Among participants with available clinical data, 25.4% reported medical comorbidities. Pain locations were heterogeneous, with lower back, neck, and shoulder pain among the most commonly reported sites.

### 3.2. Common Method Variance

Harman’s single-factor test identified six factors with eigenvalues greater than 1 in the unrotated solution. The first factor accounted for 19.50% of the total variance, and the six factors together accounted for 62.28%. This pattern did not suggest a dominant single factor under this limited diagnostic; however, Harman’s test cannot rule out common method variance [44].

### 3.3. Descriptive and Correlation Analyses

Descriptive statistics and correlations for the study variables are presented in Table 2. Pain centrality was negatively correlated with self-compassion (*r* = −0.40, *p* < 0.001) and trust in physicians (*r* = −0.17, *p* = 0.002). Self-compassion was positively correlated with trust in physicians (*r* = 0.27, *p* < 0.001). Among the covariates, age, average pain intensity during the previous 7 days, and log-transformed pain duration were positively correlated with pain centrality, whereas sex was negatively correlated with pain centrality. Pain intensity was also negatively correlated with self-compassion. These findings were consistent with the hypothesized bivariate associations among the three core variables. The small positive correlation between 7-day average pain intensity and trust in physicians was not statistically significant (*r* = 0.06, *p* = 0.275) and was therefore not interpreted further.

### 3.4. Regression Diagnostics

Visual inspection of the residual plots did not indicate serious violations of linearity, homoscedasticity, or residual normality. Across the two regression equations, variance inflation factors ranged from 1.012 to 1.349, indicating no serious multicollinearity. The maximum absolute studentized deleted residual was 3.473, the maximum leverage value was 0.069, and the maximum Cook’s distance was 0.041. Two observations had absolute studentized deleted residuals greater than 3 and were therefore examined further. No apparent data-entry errors were identified, and their Cook’s distances were small (≤ 0.022), indicating limited influence on the regression estimates. Accordingly, no cases were excluded on the basis of the regression diagnostics. Exploratory quadratic terms were nonsignificant for self-compassion in predicting pain centrality (*p* = 0.495) and trust in physicians (*p* = 0.432), and for pain centrality in predicting trust in physicians (*p* = 0.671).

### 3.5. Indirect Association Analysis

**Figure 1:**
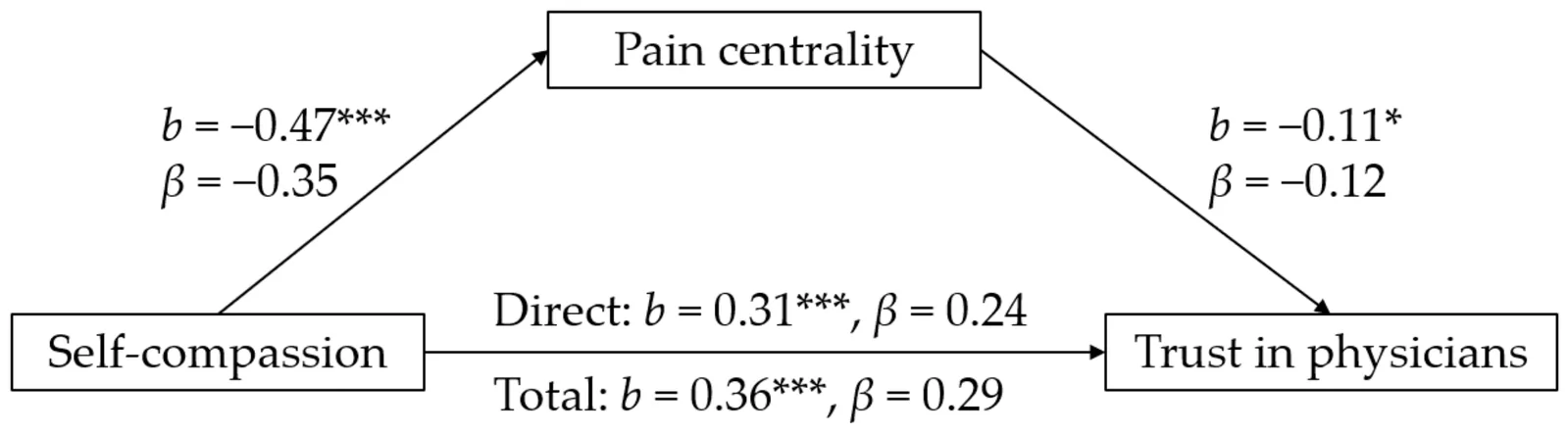
Statistical Indirect-Association Model of Trust in Physicians. Note. *b* = unstandardized regression coefficient; *β* = standardized regression coefficient. Age, sex, average pain intensity during the previous 7 days, and log-transformed pain duration were included as covariates but are not displayed. * *p* < 0.05. *** *p* < 0.001.

The hypothesized indirect association through pain centrality was tested using PROCESS Model 4 while controlling for age, sex, average pain intensity during the previous 7 days, and log-transformed pain duration. As shown in Table 3, self-compassion was positively associated with trust in physicians in the total-association model (*b* = 0.36, SE = 0.06, 95% CI [0.24, 0.49], *p* < 0.001), and negatively associated with pain centrality (*b* = −0.47, SE = 0.06, 95% CI [−0.59, −0.35], *p* < 0.001). When pain centrality was included in the outcome model, pain centrality was negatively associated with trust in physicians (*b* = −0.11, SE = 0.06, 95% CI [−0.22, −0.01], *p* = 0.040), while self-compassion remained positively associated with trust in physicians (*b* = 0.31, SE = 0.07, 95% CI [0.17, 0.45], *p* < 0.001). The final outcome model explained 10.2% of the variance in trust in physicians, and explanatory power was modest across the regression models overall (R^2^ = 0.09–0.26). The estimated indirect association through pain centrality was statistically significant (estimate = 0.054, Boot SE = 0.027, 95% CI [0.0004, 0.1093]), while the direct association also remained statistically significant (Figure 1). Thus, the primary analysis was consistent with Hypotheses 1 and 2.

### 3.6. Sensitivity Analyses

Across the sensitivity analyses, indirect-association estimates were consistently positive and ranged from 0.036 to 0.068 (Supplementary Table S1). The 95% bootstrap confidence intervals excluded zero in the primary model, the model using raw pain duration, and the models using 6-month average or worst pain intensity, whereas they included zero in the unadjusted model, the model adjusted for age and sex, and the model using current pain intensity. The indirect-association estimate was nearly unchanged when raw rather than log-transformed pain duration was used. Overall, the magnitude of the indirect association was relatively similar across model specifications, although whether the bootstrap confidence interval excluded zero was sensitive to covariate adjustment and the operationalization of pain intensity.

## 4. Discussion

Trust in physicians is important for treatment adherence and health-related behaviors [45,46]. Previous research on trust in physicians has predominantly focused on physician-related factors and patients’ demographic characteristics, whereas patients’ psychological traits (e.g., self-compassion) and their potential correlates have received less attention. The present study found that self-compassion was positively associated with trust in physicians among Chinese patients with chronic pain and that an indirect statistical association through pain centrality was also observed.

This study contributes to the literature on trust in physicians and self-compassion in several ways. First, it identifies self-compassion as a positive patient-level psychological characteristic associated with greater trust in physicians among Chinese patients with chronic pain. This finding extends research on the correlates of trust in physicians beyond demographic and experiential factors—such as age [42], ethnicity [47], and prior misdiagnosis experiences [48]—by highlighting the relevance of patients’ psychological characteristics. Although direct evidence linking self-compassion to trust in physicians remains limited, this finding is consistent with studies showing positive associations between self-compassion and interpersonal trust in adolescent and adult samples [15,16]. It also complements evidence linking physician empathy, communication, and patients’ adaptive coping with more constructive clinical relationships [24,49,50]. Future longitudinal or intervention studies could examine whether changes in self-compassion are accompanied by changes in trust in physicians.

Second, this study provides preliminary evidence extending the scope of outcomes associated with self-compassion in chronic pain beyond patients’ intrapersonal pain adjustment to a relational healthcare outcome. Previous research has largely focused on associations between self-compassion and intrapersonal adjustment outcomes, including psychological distress, pain acceptance, coping strategies, and functional status [51,52,53]. In the present study, self-compassion was also associated with greater trust in physicians. According to self-compassion theory, the common humanity component emphasizes that suffering is part of the shared human experience, which may reduce feelings of isolation and strengthen a sense of connection with others [13]. This relational orientation may also be relevant to how patients interpret interpersonal experiences in healthcare settings. However, the present study did not examine the specific contribution of common humanity or directly assess such interactional processes, so this interpretation remains theoretical.

Third, an indirect statistical association through pain centrality was observed between self-compassion and trust in physicians, suggesting that pain-related self-representation may be statistically relevant to the association between an intrapersonal psychological resource and a relational healthcare outcome. The two component associations provide context for this finding. First, self-compassion was associated with lower pain centrality. Although direct evidence linking self-compassion to pain centrality remains limited, greater self-compassion has been associated with higher pain acceptance and better functioning [21,22]. Second, pain centrality was negatively associated with trust in physicians. Direct evidence linking pain centrality to trust in physicians remains limited; however, prior research has highlighted the importance of having patients’ subjective pain experiences believed and taken seriously [25,26], documented stigma and invalidation among people with chronic pain [27,28], and associated attentive listening and perceived physician empathy with greater trust in physicians [29,30]. When pain assumes a more central position in patients’ lives, physicians’ responses to pain may therefore carry greater personal and relational significance in trust judgments. Greater pain centrality may also coincide with greater sensitivity to perceived dismissal or misunderstanding, although these interactional processes were not directly assessed in the present study. Pain centrality may thus be relevant not only to adjustment to pain but also to patients’ experiences of healthcare relationships.

Taken together, these findings highlight the potential relevance of patient-level psychological characteristics and pain-related self-representation to trust in physicians among patients with chronic pain.

Although the present study assessed trust in physicians rather than trust in nurses or nursing outcomes, the findings may have indirect relevance to nursing care for hospitalized patients with chronic pain. Future research could examine whether considering pain centrality alongside conventional pain assessment provides additional information about patients’ psychosocial experiences. Studies could also evaluate whether brief communication approaches, such as open-ended questions about how pain affects patients’ roles and sense of self or explicit validation of pain experiences, are acceptable and useful for patients with high pain centrality. The clinical utility of the Centrality of Pain Scale as a brief assessment aid should be established before routine use.

### Limitations and Future Directions

The current study has several limitations. First, participants were recruited using convenience sampling from a single tertiary hospital, which may have introduced selection bias because patients who were available and willing to participate may differ from other eligible patients. The inpatient setting may also limit the generalizability of the findings to other clinical settings and chronic pain populations. Moreover, chronic pain is clinically heterogeneous, and differences in pain conditions and clinical characteristics may affect the generalizability of the observed associations. Excluding patients with documented psychiatric disorders may further limit the generalizability to chronic pain populations with psychiatric comorbidity. Because the numbers of patients initially approached and those who declined participation were not systematically recorded, a formal response rate could not be calculated, limiting assessment of potential nonresponse bias. Future research should use more systematic sampling strategies and recruit participants across multiple clinical settings and more diverse chronic pain populations to strengthen external validity.

Second, the cross-sectional design prevents determination of the temporal ordering or causal relationships among self-compassion, pain centrality, and trust in physicians. Longitudinal and experimental studies are needed to clarify these relationships. Because no prospective power analysis specifically targeted the indirect association, its statistical precision should be interpreted from the bootstrap confidence interval. In the primary analysis, the lower bound was close to zero, and some sensitivity analyses yielded confidence intervals that included zero. The indirect association should therefore be interpreted cautiously and replicated in independent samples.

Third, the primary study constructs were assessed using self-report questionnaires, which may have introduced common method variance and influenced the magnitude of the observed associations through factors such as social desirability or affective consistency [44]. Although Harman’s single-factor test did not indicate a dominant single factor, common method variance cannot be ruled out because the primary constructs were assessed within the same session. In addition, the physician referent was not standardized, and hospitalized patients may have interpreted “my doctor” as either a particular treating physician or physicians more generally, potentially introducing heterogeneity into trust ratings. Although validated Chinese versions of the study measures were used, the psychometric properties in hospitalized Chinese adults with chronic pain warrant further evaluation. Future studies could incorporate complementary non-self-report measures where appropriate.

Fourth, although age, sex, pain intensity, and pain duration were included as covariates, residual and unmeasured confounding cannot be ruled out. Other patient- and physician-related factors, such as pain catastrophizing, depressive and anxiety symptoms, previous healthcare experiences, physician empathy [30,54], and communication quality [49], may also be relevant to the observed associations. In addition, the present study focused on a specific statistical indirect-association model involving self-compassion, pain centrality, and trust in physicians, and other psychological, relational, and healthcare-related factors may also be relevant. Future studies could examine additional variables and potential moderators, including prior medical trauma, perceived discrimination, and distrust in the healthcare system. They could also examine whether self-kindness, common humanity, and mindfulness show distinct associations with pain centrality and trust in physicians rather than assuming uniform contributions across self-compassion components.

## 5. Conclusions

This cross-sectional study found that greater self-compassion was associated with higher trust in physicians among Chinese patients with chronic pain, with an indirect statistical association observed through pain centrality. These findings are consistent with a broader biopsychosocial and person-centered understanding of chronic pain by highlighting the relevance of patients’ psychological resources and pain-related self-representations to healthcare relationships. Replication in diverse populations and longitudinal studies is needed to clarify these associations before the potential clinical utility of self-compassion and pain centrality in person-centered chronic pain assessment and care can be evaluated.

## Figures and Tables

**Table 1:** Demographic and Clinical Characteristics of Eligible Participants (*N* = 376).

Characteristic	Available *n*	Value
***Demographic characteristics***		
**Age, years, M (SD)**	376	44.00 (14.12)
**Sex, *n* (%)**	374	
Female		202 (54.0)
Male		172 (46.0)
**Marital status, *n* (%)**	373	
Unmarried		72 (19.3)
Married		281 (75.3)
Divorced		15 (4.0)
Remarried		1 (0.3)
Widowed		4 (1.1)
**Place of residence, *n* (%)**	363	
Urban		285 (78.5)
Town		36 (9.9)
Rural		42 (11.6)
**Educational attainment, *n* (%)**	374	
No formal education		8 (2.1)
Primary school		36 (9.6)
Junior high school		62 (16.6)
Senior high school/technical secondary school		75 (20.1)
College/university		173 (46.3)
Postgraduate or above		20 (5.3)
**Employment status, *n* (%)**	363	
Employed		203 (55.9)
Farming		38 (10.5)
Resigned/retired		56 (15.4)
Unemployed		35 (9.6)
Other		31 (8.5)
**Monthly personal income, RMB, *n* (%)**	368	
<1000/month		50 (13.6)
1000–3000/month		77 (20.9)
3001–5000/month		115 (31.3)
>5000/month		126 (34.2)
***Clinical characteristics***		
**Average pain intensity during previous 7 days, M (SD)**	375	4.14 (2.06)
**Pain duration, months, median (IQR)**	376	27 (13–60)
Range		3–600
**Medical comorbidity, *n* (%)**	370	
No		276 (74.6)
Yes		94 (25.4)
**Pain pattern, *n* (%)**	322	
Continuous pain		54 (16.8)
Intermittent pain		243 (75.5)
Continuous pain with exacerbations		25 (7.8)
**Pain location, *n* (%) ^a^**	375	
Head/face		34 (9.1)
Neck		104 (27.7)
Shoulder		61 (16.3)
Upper extremity (arm/hand)		27 (7.2)
Chest		7 (1.9)
Abdomen		11 (2.9)
Upper back		16 (4.3)
Lower back		147 (39.2)
Hip/gluteal region		11 (2.9)
Lower extremity (leg/foot)		41 (10.9)

***Note.*** Values are presented as *n* (%), *M* (SD), or median (IQR), as appropriate. *n* = number of participants; *M* = mean; SD = standard deviation; IQR = interquartile range. Percentages are based on available data for each characteristic; therefore, denominators vary because of missing responses. ^a^ Participants could report more than one pain location; therefore, percentages for pain location may exceed 100%.

**Table 2:** Descriptive Statistics and Correlations Among the Study Variables.

Variables	*M*	*SD*	1	2	3	4	5	6	7
Sex	0.46	0.50	—						
2. Age (years)	43.98	14.11	0.05	—					
3. 7-day average pain intensity	4.14	2.07	−0.05	0.34 ***	—				
4. Log-transformed pain duration	3.37	1.03	0.04	0.34 ***	0.19 ***	—			
5. Self-compassion	3.29	0.48	0.06	−0.07	−0.15 **	0.03	—		
6. Pain centrality	2.65	0.64	−0.11 *	0.25 ***	0.32 ***	0.14 **	−0.40 ***	—	
7. Trust in physicians	3.73	0.61	−0.09	−0.01	0.06	−0.02	0.27 ***	−0.17 **	—

***Note.*** *N* = 367. Correlations are Pearson’s *r*. Sex: 0 = female, 1 = male. Pain duration was natural-log transformed as ln(months + 1). * *p* < 0.05. ** *p* < 0.01. *** *p* < 0.001.

**Table 3:** Regression Estimates for the Primary Indirect-Association Model.

**Panel A. Regression Model: Outcome = Pain Centrality**				
**Variables**	***b***	**SE**	**95% CI**	***p***
Sex	−0.12	0.06	[−0.234, −0.002]	0.046
Age	0.0062	0.0023	[0.0017, 0.0108]	0.007
Pain intensity	0.06	0.02	[0.03, 0.09]	<0.001
Pain duration, ln(months + 1)	0.04	0.03	[−0.02, 0.10]	0.183
Self-compassion	−0.47	0.06	[−0.59, −0.35]	<0.001
Model fit: *R*^2^ = 0.26, *F* (5, 361) = 25.22, *p* < 0.001.				
**Panel B. Total-Association Model: Outcome = Trust in Physicians**				
**Variables**	***b***	**SE**	**95% CI**	***p***
Sex	−0.12	0.06	[−0.24, 0.01]	0.060
Age	−0.0001	0.0024	[−0.0049, 0.0047]	0.978
Pain intensity	0.03	0.02	[−0.001, 0.062]	0.057
Pain duration, ln(months + 1)	−0.03	0.03	[−0.09, 0.04]	0.385
Self-compassion	0.36	0.06	[0.24, 0.49]	<0.001
Model fit: *R*^2^ = 0.09, *F* (5, 361) = 7.23, *p* < 0.001.				
**Panel C. Direct-Association Model: Outcome = Trust in Physicians**				
**Variables**	***b***	**SE**	**95% CI**	***p***
Sex	−0.13	0.06	[−0.25, −0.01]	0.036
Age	0.0006	0.0024	[−0.0042, 0.0055]	0.792
Pain intensity	0.04	0.02	[0.01, 0.07]	0.021
Pain duration, ln(months + 1)	−0.02	0.03	[−0.09, 0.04]	0.467
Self-compassion	0.31	0.07	[0.17, 0.45]	<0.001
Pain centrality	−0.11	0.06	[−0.22, −0.01]	0.040
Model fit: *R*^2^ = 0.10, *F* (6, 360) = 6.78, *p* < 0.001.				

***Note.*** *N* = 367. Coefficients are unstandardized. Sex: 0 = female, 1 = male. Pain intensity refers to average pain intensity during the previous 7 days. Pain duration was natural-log transformed as ln(months + 1).

## Data Availability

The data presented in this study are available from the corresponding author upon reasonable request. The data are not publicly available because the informed consent obtained from participants stated that all collected data would be kept strictly confidential and used only for research purposes, and did not include consent for the public sharing or deposition of individual-level data.

## Supplementary Materials

The following supporting information can be downloaded at: https://www.mdpi.com/article/10.3390/healthcare14203383/s1, Table S1: Sensitivity analyses of the indirect association through pain centrality.
