# Supplementary material for: Self-Compassion, Pain Centrality, and Trust in Physicians Among Chinese Patients with Chronic Pain: A Cross-Sectional Study
Source: Healthcare (Basel). 2026 Oct 10;14(20):3383. doi: 10.3390/healthcare14203383 (PMC13704551; doi:10.3390/healthcare14203383)
Supplement: Supplementary file 1 [file healthcare-14-03383-s001.zip › healthcare-4516956-supplementary.pdf]

Supplementary Table S1

Sensitivity Analyses of the Indirect Association Through Pain Centrality

Model	Model specification	Indirect-association estimate	Boot SE	95% bootstrap CI
A	No covariates	0.036	0.029	[−0.020, 0.094]
B	Age and sex	0.044	0.027	[−0.010, 0.099]
C	Age, sex, 7-day average pain intensity, and log-transformed pain duration (primary model)	0.054	0.027	[0.0004, 0.1093]
D	Age, sex, 7-day average pain intensity, and raw pain duration	0.053	0.027	[0.001, 0.108]
E	Age, sex, current pain intensity, and log-transformed pain duration	0.048	0.026	[−0.003, 0.101]
F	Age, sex, 6-month average pain intensity, and log-transformed pain duration	0.064	0.027	[0.013, 0.118]
G	Age, sex, 6-month worst pain intensity, and log-transformed pain duration	0.068	0.030	[0.010, 0.127]

Note. $N = 367$. Self-compassion was specified as X, pain centrality as M, and trust in physicians as Y. Confidence intervals were based on 5,000 bootstrap samples. Log-transformed pain duration was calculated as ln(pain duration + 1).
